# Association of Weight for Length vs Body Mass Index During the First 2 Years of Life With Cardiometabolic Risk in Early Adolescence

**DOI:** 10.1001/jamanetworkopen.2018.2460

**Published:** 2018-09-21

**Authors:** Izzuddin M. Aris, Sheryl L. Rifas-Shiman, Ling-Jun Li, Seungmi Yang, Mandy B. Belfort, Jennifer Thompson, Marie-France Hivert, Rita Patel, Richard M. Martin, Michael S. Kramer, Emily Oken

**Affiliations:** 1 Singapore Institute for Clinical Sciences, Agency for Science, Technology and Research, Singapore, Singapore; 2 Division of Chronic Disease Research Across the Lifecourse, Department of Population Medicine, Harvard Medical School and Harvard Pilgrim Health Care Institute, Boston, Massachusetts; 3 Department of Obstetrics and Gynecology, Yong Loo Lin School of Medicine, National University of Singapore, Singapore, Singapore; 4 Division of Obstetrics and Gynecology, KK Women’s and Children’s Hospital, Singapore, Singapore; 5 Obstetrics and Gynecology Academic Clinical Programme, Duke–National University of Singapore Graduate Medical School, Singapore, Singapore; 6 Singapore Eye Research Institute, Singapore National Eye Centre, Singapore, Singapore; 7 Department of Pediatrics, Faculty of Medicine, McGill University, Montreal, Quebec, Canada; 8 Department of Epidemiology, Biostatistics, and Occupational Health, Faculty of Medicine McGill University, Montreal, Quebec, Canada; 9 Department of Pediatric Newborn Medicine, Brigham and Women's Hospital, Boston, Massachusetts; 10 Diabetes Unit, Massachusetts General Hospital, Boston; 11 School of Social and Community Medicine, University of Bristol, Bristol, United Kingdom; 12 Department of Nutrition, Harvard T.H. Chan School of Public Health, Boston, Massachusetts

## Abstract

**Importance:**

The American Academy of Pediatrics currently recommends weight for length (WFL) for assessment of weight status in children younger than 2 years but body mass index (BMI) for children older than 2 years. However, the clinical implications of using WFL vs BMI in children younger than 2 years as an indicator of future health outcomes remains understudied.

**Objective:**

To compare associations of overweight based on WFL vs BMI in children younger than 2 years with cardiometabolic outcomes during early adolescence.

**Design, Setting, and Participants:**

This prospective study of birth cohorts in the United States (Project Viva) and Belarus (Promotion of Breastfeeding Intervention Trial [PROBIT]) performed from June 1, 1996, to November 31, 2002, included 13 666 children younger than 2 years.

**Main Exposures:**

Overweight defined as Centers for Disease Control and Prevention (CDC) WFL in the 95th percentile or greater, World Health Organization (WHO) WFL in the 97.7th percentile or greater, or WHO BMI in the 97.7th percentile or greater at 6, 12, 18, or 24 months of age.

**Main Outcomes and Measures:**

Primary outcomes were fat mass index, insulin resistance, metabolic risk score, and obesity during early adolescence. Secondary outcomes were height and BMI *z* scores, sum of skinfolds, waist circumference, and systolic blood pressure during early adolescence.

**Results:**

The study included 919 children (mean [SD] age, 12.9 [0.9] years; 460 [50.1%] male; and 598 [65.1%] white) from Project Viva and 12 747 children (mean [SD] age, 11.5 [0.5] years; 6204 [48.7%] male; and 12 747 [100%] white) from PROBIT. During 6 to 24 months of age, in Project Viva, 206 children (22.4%) were overweight at any of the 4 times points according to the CDC WFL, 160 (17.4%) according to WHO WFL, and 161 (17.5%) according to WHO BMI cut points. In PROBIT, 3715 children (29.1%) were overweight at any of the 4 time points according to the CDC WFL, 3069 (24.1%) according to WHO WFL, and 3125 (24.5%) according to WHO BMI cut points. After maternal and child characteristics were adjusted for, being ever overweight (vs never overweight) during 6 to 24 months of age was associated with higher likelihood of adverse cardiometabolic risk markers during early adolescence, but associations did not differ substantially across WFL and BMI cut points in either cohort. For example, for fat mass index in Project Viva, β = 0.9 (95% CI, 0.5-1.4) for the CDC WFL, β = 1.1 (95% CI, 0.6-1.6) for WHO WFL, and β = 1.4 (95% CI, 0.9-1.9) for WHO BMI. For PROBIT, β = 0.5 (95% CI, 0.4-0.6) for the CDC WFL, β = 0.6 (95% CI, 0.5-0.7) for WHO WFL, and β = 0.6 (95% CI, 0.5-0.6) for WHO BMI. Neither growth metric in infancy was superior over the others based on *F* statistics (Project Viva: 17.1-17.8; PROBIT: 87.1-88.7). Findings were similar for insulin resistance, metabolic risk score, obesity, and secondary outcomes.

**Conclusions and Relevance:**

Choice of WFL vs BMI to define overweight during the first 2 years of life may not greatly affect the association with cardiometabolic outcomes during early adolescence. The findings appear to have important implications for investigators seeking to use BMI as a growth metric for epidemiologic research and for practitioners monitoring the weight status of children younger than 2 years.

## Key Points

**Question:**

Is weight for length or body mass index in children younger than 2 years a better indicator of future health outcomes?

**Findings:**

In this study of 2 birth cohorts, being ever overweight (vs never overweight) during 6 to 24 months of age provided adjusted estimates for early adolescent cardiometabolic outcomes that did not differ substantially across weight for length and body mass index cut points.

**Meaning:**

Choice of weight for length vs body mass index to define overweight during the first 2 years of life may not greatly affect the association with cardiometabolic outcomes during early adolescence.

## Teaser

This study of birth cohorts from the United States and Belarus examines whether weight for length or body mass index more accurately estimates future cardiometabolic outcomes in children younger than 2 years.

## Introduction

Physical growth of children is a well-recognized indicator of subsequent health and wellness.^1,2^ The American Academy of Pediatrics and the Centers for Disease Control and Prevention (CDC) currently recommend using weight for length (WFL) for assessment of overweight in children younger than 2 years^3^; WFL is also a predominant standard used internationally.^4^ However, WFL percentile curves do not reflect the age-dependent variation of weight or length with age. The World Health Organization (WHO) has provided body mass index (BMI) (calculated as weight in kilograms divided by height in meters squared) for age curves for children 0 to 5 years of age, which overcomes this limitation.^5^

Because the CDC^6^ and WHO^5^ charts are available, practitioners and researchers now have a choice of growth charts and anthropometric measures to use. It is therefore important to understand how they compare in estimating later clinical outcomes because practitioners might prefer to use the metric that more strongly indicates later health. However, to date, few studies have compared these anthropometric measures in terms of their associations with direct measures of adiposity and cardiometabolic risk later in life. Rifas-Shiman et al^7^ previously reported that WFL and BMI cut points for overweight during the first 2 years of life provided similar estimates of obesity risk at 5 years. Roy et al^8^ observed that high BMI at 2 to 6 months of age was more strongly associated with obesity at 2 years than was high WFL. To our knowledge, no studies have compared being overweight by WFL or BMI percentiles during the first 2 years of life in association with later adiposity or other cardiometabolic risk markers other than BMI, such as insulin resistance or metabolic risk score. To address the gaps in the literature, we used data from 2 longitudinal cohorts (Project Viva^9^ and the Promotion of Breastfeeding Intervention Trial [PROBIT]^10^) to compare associations of being overweight by the CDC WFL, WHO WFL, or WHO BMI cut points during the first 2 years of life with cardiometabolic outcomes during early adolescence. Analyzing data in 2 different populations with different confounding structures enabled us to assess the robustness of the observed associations. We hypothesized that being overweight during the first 2 years by any of the 3 cut points would provide similar estimates of association.

## Methods

The study was performed from June 1, 1996, to November 31, 2002. The institutional review board of Harvard Pilgrim Health Care approved Project Viva in line with ethical standards established by the Declaration of Helsinki.^11^ The initial PROBIT and subsequent follow-ups were approved by the Belarussian Ministry of Health and received ethical approval from the McGill University Health Centre Research Ethics Board, the institutional review board at Harvard Pilgrim Health Care, and the Avon Longitudinal Study of Parents and Children Law and Ethics Committee. This study followed the Strengthening the Reporting of Observational Studies in Epidemiology (STROBE) reporting guideline.

### Study Populations 

Project Viva is an ongoing prospective cohort study of prenatal and perinatal influences on maternal, fetal, and child health, as detailed elsewhere.^9^ Mothers provided written informed consent at enrollment and follow-up visits, and children provided verbal informed assent during the early adolescent visit. All data were deidentified.

During research examinations during infancy (median age, 6.3 months; age range, 4.9-10.6 months), trained research assistants measured weight and length using standardized protocols.^12,13^ We obtained additional data on weight and length from medical records, in which pediatric clinics recorded these measures at each well-child care visit during infancy and early childhood (<2 years of age). As described previously,^14^ practitioners at pediatric clinics used the paper-and-pencil technique to measure recumbent length for infants 0 to 2 years of age. We applied a correction algorithm to account for the systematic overestimation of lengths that resulted from this technique.^14^ Of 2128 live singleton births, we included 919 children (43.2%) who had a measure of weight and length at 6, 12, 18, or 24 months (within ±2 months at each time point) and at least 1 outcome measure during the early adolescent visit (median age, 12.9 years; age range, 11.9-16.6 years) (eFigure in the Supplement).

PROBIT was a cluster-randomized trial of breastfeeding promotion intervention in the Republic of Belarus. The design of PROBIT has been published previously.^15^ A parent or legal guardian provided written informed consent in Russian at enrollment and at the follow-up visits, and all children provided written informed assent during the 11.5-year visit. All data were deidentified.

Polyclinic pediatricians measured infant weight and length with a length board during follow-up visits at 1, 2, 3, 6, 9, and 12 months of age; home visits were made when polyclinic visits were missed.^15^ We obtained additional data from polyclinic medical records, in which the study pediatricians recorded length, height, and weight data at each well-child care visit between 12 months and 6.5 years of age.^10^ We had no information on the method of length measurement in the data extracted from the medical records, but we have no reason to think that the pediatricians changed their method of length measurement until upright height measurement replaced supine length. Of 17 046 healthy, singleton, term live births, we studied 12 747 children (74.8%) who had a measure of weight and length at 6, 12, 18, or 24 months of age (within ±2 months at each time point) and at least 1 outcome measure during the early adolescent visit (median age, 11.5 years; age range, 10.2-14.5 years) (eFigure in the Supplement).

### Infant and Child Overweight Status

In both cohorts, we used length and weight measurements at 6, 12, 18, and 24 months of age (within ±2 months at each time point) to derive sex-specific CDC WFL, WHO WFL, and age- and sex-specific WHO BMI percentiles. The main exposures were being overweight at any of the 4 time points (ever overweight) between 6 and 24 months of age using each of the 3 cut points: CDC WFL 95th percentile or greater, WHO WFL 97.7th percentile or greater, or BMI 97.7th percentile or greater. In secondary analyses, we examined overweight status at each time point between 6 and 24 months of age. We also categorized children according to the number of time points at which they were overweight between 6 and 24 months of age (range, 0-4) for each of the 3 cut points. Because few children were overweight at all 4 time points, we combined those children with the children who were overweight at any 3 time points.

### Early Adolescent Body Composition and Cardiometabolic Risk Markers

At a mean of 12.9 years of age (age range, 11.9-16.6 years) in Project Viva and 11.5 years of age (age range, 10.2-14.5 years) in PROBIT, we obtained the measures of body composition and cardiometabolic risk markers.

#### Body Composition

As detailed previously, trained research assistants (in Project Viva^16,17^) and pediatricians (in PROBIT^10,18,19^) measured child’s weight, standing height, waist circumference (WC), subscapular (SS) and triceps (TR) skinfolds, and foot-to-foot bioimpedance fat mass. In both cohorts, we calculated BMI, derived age- and sex-specific height and BMI *z* scores using CDC reference data,^6^ and defined obesity as BMI in the 95th percentile or greater (vs BMI in the <95th percentile) in accordance with current guidelines of the American Academy of Pediatrics.^20^ We calculated the sum of the SS and TR skinfolds and fat mass index (FMI) (calculated as fat mass divided by height in meters squared).

#### Cardiometabolic Risk Markers

Trained research assistants (in Project Viva) and pediatricians (in PROBIT) measured the child’s systolic blood pressure (SBP) using calibrated automated oscillometric monitors, as detailed previously.^13,21^ We calculated age-, sex-, and height-specific SBP *z* scores according to the 2017 American Academy of Pediatrics blood pressure reference for adolescents.^22^ In both cohorts, we obtained blood specimens after a minimum 8-hour fast^23,24^ and measured glucose and insulin levels as detailed previously,^19,21,25^ calculated insulin resistance using the homeostasis model assessment of insulin resistance (HOMA-IR), and transformed the HOMA-IR values using natural logarithms to normalize the distribution. Furthermore, we measured high-density lipoprotein cholesterol (HDL-C) and triglyceride levels in Project Viva and apolipoprotein A-I level in PROBIT, according to standard protocols.^21,25^ We calculated cohort-specific metabolic risk *z* scores using the following variables: the mean within-cohort age- and sex-specific WC, SBP, triglyceride level (log transformed), HDL-C level (inverted), and HOMA-IR value (log transformed) in Project Viva^25^ and the mean age- and sex-specific WC, SBP, apolipoprotein A-I level, fasting insulin level, and glucose level in PROBIT.^26^ This cluster of factors that comprised the metabolic risk score were first formalized by the WHO^27^ and the National Cholesterol Education Program Adult Treatment Panel III.^28^ Previous studies by Morrison et al^29,30^ demonstrated that children with high metabolic risk scores have an increased risk of developing type 2 diabetes, cardiovascular disease, and metabolic syndrome during adulthood, suggesting that the score is important for children. In PROBIT, cardiometabolic risk markers were measured using frozen dried bloodspots. Neither triglyceride nor HDL-C level could be validly measured from those samples, and apolipoprotein A-I level was therefore used as a surrogate for dyslipidemia as previously defined by Bachorik et al.^31^

### Statistical Analysis

We assessed for agreement of overweight by CDC WFL, WHO WFL, and WHO BMI cut points using κ statistics. We used multivariable linear (for continuous outcomes) and logistic regression (for obesity) to examine associations between being ever overweight (vs never overweight) during the first 2 years of life and cardiometabolic outcomes during early adolescence, adjusting for the following covariates in each cohort: maternal age (<20, 20-34, or ≥35 years), marital status (married or cohabitating vs not married), educational attainment (nonuniversity vs university educated), prepregnancy BMI, total gestational weight gain, smoking history (never, smoked before pregnancy, or smoked during pregnancy), glucose tolerance status (normoglycemia, isolated hyperglycemia, impaired glucose tolerance, or gestational diabetes), gestational hypertensive disorders (normal blood pressure, gestational hypertension, chronic hypertension, and preeclampsia), gestational age at delivery, child race/ethnicity (white, black, Hispanic, Asian, or other), sex (male or female), birth weight for gestational age *z* scores, breastfeeding status at 6 months (formula only, weaned, mixed feeding, or breastmilk only), and age at outcome measurement for Project Viva and maternal age (<20, 20-34, or ≥35 years), maternal BMI at 6.5 years (as a proxy for BMI before pregnancy), educational attainment (did not complete or completed university), marital status (registered or unregistered marriage vs unmarried), smoking during pregnancy (yes or no), child gestational age at delivery, sex (male or female), birth weight for gestational age *z* scores, and age at outcome measurement for PROBIT.

We also assessed associations of overweight at each time point (6, 12, 18, and 24 months of age) or the number of time points with overweight with adiposity and cardiometabolic risk during early adolescence in both cohorts. For all analyses of PROBIT data, we accounted for clustered measurements within hospitals and polyclinics by including a random-effect term for hospital and polyclinic but did not adjust for intervention vs control arms because earlier analyses found no differences in early adolescent cardiometabolic outcomes between these 2 study arms.^19,21^

To compare CDC WFL, WHO WFL, and WHO BMI as predictors of adiposity and cardiometabolic outcomes, we used the overall *F* statistic from linear models estimating each of these outcomes.^32^ At each time point within each cohort, the models for CDC WFL, WHO WFL, and WHO BMI contained the same number of covariates. Thus, models with larger *F* statistics were better estimators of outcomes than models that contained a different growth metric. Because *F* statistics have little interpretational value, in accordance with Kleinman et al,^32^ we set a threshold of 5% or greater for the difference in *F* statistic values to indicate a meaningful advantage for the model with the larger value. In our study, the use of language around prediction connotes a temporal association that the exposure (ie, overweight by CDC or WHO growth metrics during 6-24 months of age) precedes the subsequent outcome and does not refer to prediction modeling. We analyzed all data using Stata, version 15 (StataCorp), conducted all statistical analyses as 2-sided, and defined statistical significance at α = .05 (*P* < .05).

## Results

Table 1 gives the characteristics of participating children from both cohorts. The study included 919 children (mean [SD] age, 12.9 [0.9] years; 460 [50.1%] male; and 598 [65.1%] white) from Project Viva and 12 747 children (mean [SD] age, 11.5 [0.5] years; 6204 [48.7%] male; and 12 747 [100%] white) from PROBIT. During 6 to 24 months of age, in Project Viva, 206 children (22.4%) were overweight at any of the 4 times points according to the CDC WFL, 160 (17.4%) according to WHO WFL, and 161 (17.5%) according to WHO BMI cut points. In PROBIT, 3715 children (29.1%) were overweight at any of the 4 time points according to the CDC WFL, 3069 (24.1%) according to WHO WFL, and 3125 (24.5%) according to WHO BMI cut points. In both cohorts, overweight children had a higher birth weight for gestational age *z* score and were more likely to have mothers who smoked during pregnancy. During early adolescence, children in Project Viva generally had higher adiposity than did children in PROBIT (Table 2).

**Table 1.** Characteristics of Study Participants in Project Viva and PROBIT^a^

Characteristic	All	Ever Overweight by WHO BMI	Never Overweight by WHO BMI	*P* Value
**Project Viva (n = 919)**				
Maternal				
Age, y				
<20	29 (3.2)	4 (2.5)	25 (3.3)	.70
20-34	610 (66.4)	111 (68.9)	499 (65.8)	
≥35	280 (30.5)	46 (28.6)	234 (30.9)	
Educational level				
Not university educated	261 (28.4)	58 (36.0)	203 (26.8)	.02
University educated	658 (71.6)	103 (64.0)	555 (73.2)	
Marital status				
Married or cohabitating	851 (92.6)	145 (90.1)	706 (93.1)	.18
Not married	68 (7.4)	16 (9.9)	52 (6.9)	
Maternal smoking history				
Never smoked	650 (70.7)	105 (65.2)	545 (71.9)	.02
Smoked before pregnancy	184 (20.0)	32 (19.9)	152 (20.1)	
Smoked during pregnancy	85 (9.3)	24 (14.9)	61 (8.1)	
Prepregnancy BMI, mean (SD)	24.8 (5.2)	25.9 (6.0)	24.6 (5.0)	.003
Total gestational weight gain, mean (SD), kg	15.6 (5.3)	16.2 (5.7)	15.4 (5.2)	.11
Glucose tolerance status				
Normoglycemia	764 (83.1)	142 (88.2)	622 (82.1)	.04
Isolated hyperglycemia	80 (8.7)	8 (5.0)	72 (9.5)	
Intermediate glucose intolerance	29 (3.2)	1 (0.6)	28 (3.7)	
Gestational diabetes	46 (5.0)	10 (6.2)	36 (4.8)	
Hypertensive disorders of pregnancy				
Normal blood pressure	822 (89.4)	142 (88.2)	680 (89.7)	.73
Gestational hypertension	63 (6.9)	11 (6.8)	52 (6.9)	
Chronic hypertension	10 (1.1)	3 (1.9)	7 (0.9)	
Preeclampsia	24 (2.6)	5 (3.1)	19 (2.5)	
Child				
Sex				
Male	460 (50.1)	90 (55.9)	370 (48.8)	.10
Female	459 (49.9)	71 (44.1)	388 (51.2)	
Race/ethnicity				
White	598 (65.1)	98 (60.9)	500 (66.0)	.04
Black	143 (15.6)	38 (23.6)	105 (13.9)	
Hispanic	39 (4.2)	6 (3.7)	33 (4.4)	
Asian	27 (2.9)	3 (1.9)	24 (3.2)	
Other	112 (12.2)	16 (9.9)	96 (12.7)	
Gestational age at delivery, mean (SD), wk	39.6 (1.6)	39.5 (1.7)	39.6 (1.6)	.42
Birth weight for gestational age *z* score, mean (SD),	0.2 (1.0)	0.4 (0.9)	0.2 (1.0)	.002
Breastfeeding status at 6 mo				
Formula only	79 (9.1)	13 (8.7)	66 (9.2)	.003
Weaned	312 (35.9)	70 (47.0)	242 (33.6)	
Mixed feeding	239 (27.5)	41 (27.5)	198 (27.5)	
Breastmilk only	239 (27.5)	25 (16.8)	214 (29.7)	
**PROBIT (n = 12 747)**				
Maternal				
Age, y				
<20	1704 (13.6)	447 (14.6)	1257 (13.3)	.20
20-34	10 284 (82.1)	2491 (81.1)	7793 (82.4)	
≥35	542 (4.3)	134 (4.4)	408 (4.3)	
Educational level				
Did not complete university	11 047 (86.7)	2718 (87.0)	8329 (86.6)	.90
Completed university	1700 (13.3)	407 (13.0)	1293 (13.4)	
Marital status				
Registered or unregistered marriage	12 280 (96.3)	3027 (96.9)	9253 (96.2)	.19
Unmarried	467 (3.7)	98 (3.1)	369 (3.8)	
Smoking during pregnancy				
No	12 499 (98.1)	3068 (98.2)	9431 (98.0)	.57
Yes	248 (1.9)	57 (1.8)	191 (2.0)	
BMI at 6.5 y	24.5 (4.4)	24.9 (4.3)	24.3 (4.4)	<.001
Child				
Sex				
Male	6204 (48.7)	1191 (38.1)	5013 (52.1)	<.001
Female	6543 (51.3)	1934 (61.9)	4609 (47.9)	
Gestational age at delivery, mean (SD), wk	39.4 (1.0)	39.4 (1.0)	39.4 (1.0)	.40
Birth weight for gestational age *z* score, mean (SD)	0.4 (1.0)	0.6 (1.0)	0.4 (1.0)	<.001

Abbreviations: BMI, body mass index (calculated as weight in kilograms divided by height in meters squared); PROBIT, Promotion of Breastfeeding Intervention Trial; WHO, World Health Organization.

^a^ Data are presented as number (percentage) of study participants unless otherwise indicated.

**Table 2.** Prevalence of Overweight at 6 to 24 Months of Age and Distributions of Early Adolescent Cardiometabolic Outcomes in Project Viva and PROBIT^a^

**Variable**	Project Viva (n = 919)^b^	PROBIT (n = 12 747)^b^
**Overweight Prevalence at the Age of 6-24 mo**		
Age of 6 mo (age range, 4-8 mo)		
CDC WFL ≥95th percentile	134/891 (15.0)	1521/12 594 (12.1)
WHO WFL ≥97.7th percentile	86/891 (9.7)	998/12 594 (7.9)
WHO BMI ≥97.7th percentile	75/891 (8.4)	895/12 594 (7.1)
Age of 12 mo (age range, 10-14 mo)		
CDC WFL ≥95th percentile	64/701 (9.1)	2594/12 707 (20.4)
WHO WFL ≥97.7th percentile	50/701 (7.1)	2180/12 707 (17.2)
WHO BMI ≥97.7th percentile	54/701 (7.7)	2171/12 707 (17.1)
Age of 18 mo (age range, 16-20 mo)		
CDC WFL ≥95th percentile	68/634 (10.7)	424/2965 (14.3)
WHO WFL ≥97.7th percentile	63/634 (9.9)	396/2965 (13.4)
WHO BMI ≥97.7th percentile	67/634 (10.6)	454/2965 (15.3)
Age of 24 mo (age range, 22-26 mo)		
CDC WFL ≥95th percentile	53/563 (9.4)	628/4590 (13.7)
WHO WFL ≥97.7th percentile	48/563 (8.5)	567/4590 (12.3)
WHO BMI ≥97.7th percentile	44/563 (7.8)	628/4590 (13.7)
Ever overweight		
CDC WFL ≥95th percentile	206/919 (22.4)	3715/12 747 (29.1)
WHO WFL ≥97.7th percentile	160/919 (17.4)	3069/12 747 (24.1)
WHO BMI ≥97.7th percentile	161/919 (17.5)	3125/12 747 (24.5)
**Early Adolescent Outcomes**		
Height *z* score, mean (SD)	0.3 (1.0)	0.3 (1.0)
BMI *z* score, mean (SD)	0.4 (1.1)	−0.1 (1.0)
Sum of skinfolds, mean (SD), mm	28.4 (13.9)	23.1 (11.4)
FMI, mean (SD)	4.9 (3.5)	3.3 (2.2)
Waist circumference, mean (SD), cm	73.0 (11.8)	64.8 (8.1)
SBP *z* score, mean (SD)	−0.1 (0.8)	0.4 (0.8)
HOMA-IR value, median (IQR)	2.6 (2.1)	0.9 (1.2)
HDL-C level, mean (SD), mg/dL	55.2 (13.6)	NA
Triglycerides level, mean (SD), mg/dL	70.3 (31.1)	NA
Apolipoprotein A-I level, mean (SD), mg/dL	NA	160 (40)
Metabolic risk score, mean (SD)	−0.02 (0.6)	0.01 (0.5)
Obesity	113 (12.3)	632 (5.0)

Abbreviations: BMI, body mass index (calculated as weight in kilograms divided by height in meters squared); CDC, Centers for Disease Control and Prevention; FMI, fat mass index (calculated as fat mass divided by height in meters squared); HDL-C, high-density lipoprotein cholesterol; HOMA-IR, homeostasis model assessment of insulin resistance; IQR, interquartile range; NA, not applicable; PROBIT, Promotion of Breastfeeding Intervention Trial; SBP, systolic blood pressure; WFL, weight for length; WHO, World Health Organization.

SI conversion factors: To convert apolipoprotein A-I to grams per liter, multiply by 0.01; HDL-C to millimoles per liter, multiply by 0.0259; and triglycerides to millimoles per liter, multiply by 0.0113.

^a^ Data are presented as number (percentage) of study participants/total number unless otherwise indicated.

^b^ Sample size is based on children who had a measure of weight and length at 6, 12, 18, or 24 months of age (within ±2 months at each time point) and at least 1 outcome measure at the early adolescent visit.

We observed strong intraclass correlations between the CDC and WHO *z* scores and agreements among overweight by the CDC WFL, WHO WFL, and WHO BMI cut points in both cohorts (eTable 1 in the Supplement). In Project Viva, we observed no differences in overweight prevalence between included and excluded children. Children included in the study, however, were more likely to have mothers who were older (≥35 years), were university educated, and had breastfed at 6 months but were less likely to have mothers who smoked during pregnancy compared with children who were excluded. In PROBIT, the differences in characteristics and overweight prevalence between included and excluded children were small overall (eTable 2 in the Supplement).

**Figure 1.**
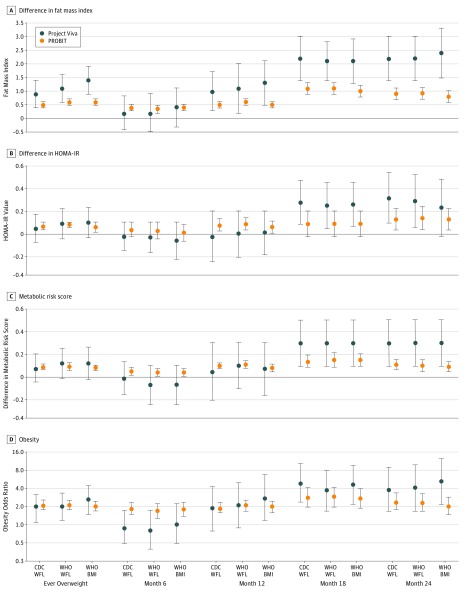
Overweight Status at 6 to 24 Months of Age Compared With Children Who Were Not Overweight Covariates adjusted for are described in the Statistical Analysis subsection of the Methods section. Fat mass index is calculated as fat mass divided by height in meters squared. Error bars represent 95% CIs. BMI indicates body mass index; CDC, Centers for Disease Control and Prevention; HOMA-IR, homeostasis model assessment of insulin resistance; PROBIT, Promotion of Breastfeeding Intervention Trial; WFL, weight for length; and WHO, World Health Organization.

In both cohorts, we found that ever overweight (vs never overweight) at any of the 4 time points during the first 2 years of life was associated with higher FMI (Figure 1A), BMI *z* score, sum of SS and TR skinfolds, and WC (eTable 3 in the Supplement) during early adolescence. Significant associations with higher HOMA-IR value and metabolic risk *z* score were observed only in PROBIT (Figure 1B and C), perhaps because of the relatively small number of Project Viva children with fasting blood samples and thus limited power in that population. No associations with HDL-C or triglyceride levels were evident. Ever overweight at 6 to 24 months of age was associated with unadjusted (eTable 3 in the Supplement) and adjusted estimates for early adolescent FMI, HOMA-IR value, metabolic risk *z* score, and odds of obesity (Figure 1) that did not differ substantially across WFL and BMI cut points. In Project Viva, the adjusted estimates and *F* statistics for FMI were β = 0.9 (95% CI, 0.5-1.4) and *F* = 17.1 for the CDC WFL, β = 1.1 (95% CI, 0.6-1.6) and *F* = 17.3 for WHO WFL, and β = 1.4 (95% CI, 0.9-1.9) and *F* = 17.8 for WHO BMI. No interactions were observed between race/ethnicity and overweight status for any outcomes in Project Viva. In PROBIT, the adjusted estimates for FMI were similar in direction but of lower magnitude: β = 0.5 (95% CI, 0.4-0.6) and *F* = 88.7 for the CDC WFL, β = 0.6 (95% CI, 0.5-0.7) and *F* = 88.3 for WHO WFL, and β = 0.6 (95% CI, 0.5-0.6) and *F* = 87.1 for WHO BMI. When comparing the *F* statistics, we observed that none of the metrics was superior (≥5% larger) to the others. Similar findings were observed for other outcomes (eTable 3 in the Supplement).

In both cohorts, we observed adjusted estimates of association with adolescent FMI (Figure 1A), BMI *z* score, sum of SS and TR skinfolds, WC (eTables 4-7 in the Supplement), HOMA-IR value (Figure 1B), metabolic risk *z* score (Figure 1C), and odds of obesity (Figure 1D) during early adolescence that were generally higher with increasing age (from 6 to 24 months) at overweight. No associations with HDL-C or triglyceride levels were observed, and we did not see any interactions in Project Viva between race/ethnicity and overweight status for all outcomes. Choice of WFL or BMI to define overweight at each time point between 6 and 24 months of age did not greatly affect the unadjusted (eTables 4-7 in the Supplement) or adjusted estimates of associations with FMI, HOMA-IR value, metabolic risk z score, and odds of obesity (Figure 1). When comparing the *F* statistics, neither growth metric was superior to the others. Similar findings were observed for other outcomes (eTables 4-7 in the Supplement).

**Figure 2.**
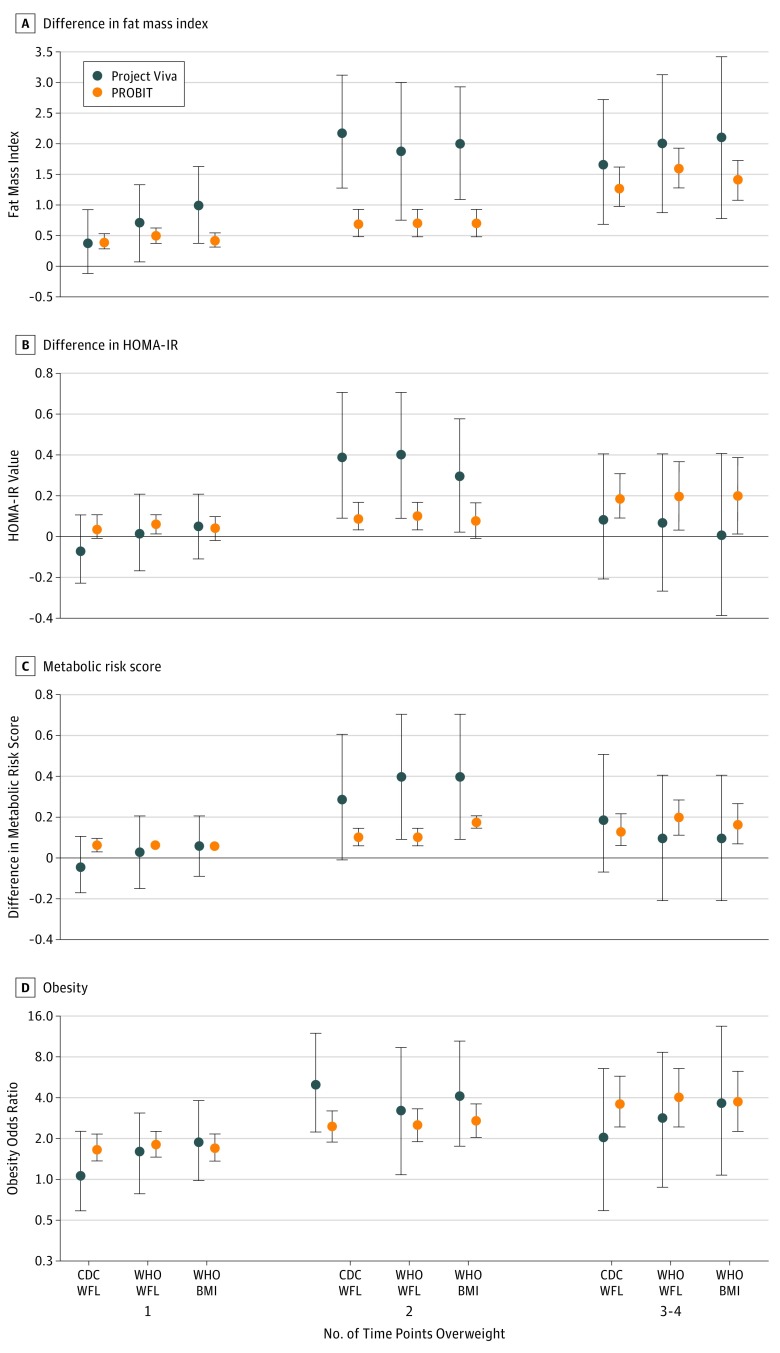
Number of Time Points Overweight Between 6 and 24 Months of Age Covariates adjusted for are described in the Statistical Analysis subsection of the Methods section. Fat mass index is calculated as fat mass divided by height in meters squared. Error bars represent 95% CIs. BMI indicates body mass index; CDC, Centers for Disease Control and Prevention; HOMA-IR, homeostasis model assessment of insulin resistance; PROBIT, Promotion of Breastfeeding Intervention Trial; WFL, weight for length; and WHO, World Health Organization.

In PROBIT, each additional time point from 6 to 24 months of age at which the child was overweight was associated with increasing FMI (Figure 2A), BMI *z* score, sum of SS and TR skinfolds, WC (eTable 8 in the Supplement), HOMA-IR value (Figure 2B), metabolic risk *z* score (Figure 2C), and odds of obesity (Figure 2D) during early adolescence. For example, the adjusted FMI estimate for overweight at 2 vs 0 time points during 6 to 24 months of age was 0.7 (95% CI, 0.6-0.9) using WHO BMI, whereas for overweight at 3 to 4 time points, the adjusted estimate was 1.4 (95% CI, 1.1-1.7). In Project Viva, each additional time point that the child was overweight during 6 to 24 months of age was associated with increasing FMI (Figure 2A) during early adolescence, whereas point estimates for HOMA-IR value and metabolic risk *z* score (Figure 2B and C) were higher in children who were overweight at 2 time points compared with 0 time points. For both cohorts, estimates that used the CDC or WHO cut points were similar (eTable 8 in the Supplement).

## Discussion

We found that being ever overweight during the first 2 years of life was an indicator of higher fat-free mass and adiposity during early adolescence. Consistent with previous findings,^33^ the CDC WFL cut point classified more children as overweight than did the WHO WFL or BMI cut points, which is not surprising given that the CDC charts used a lower percentile cut point than did the WHO charts for classifying overweight.^5,6^ Of more importance, the estimates of association with FMI, insulin resistance, and metabolic risk score during early adolescence did not differ greatly among the 3 cut points.

Our findings suggest that choice of WFL or BMI in children younger than 2 years does not substantially affect the ability to estimate future adiposity and cardiometabolic outcomes. Earlier studies^8,34^ have identified high concordance between WFL and BMI after 6 months of age, indicating that either metric may be a reasonable measure during later infancy for assessing risk of later health outcomes. However, BMI may be preferable to WFL for other reasons. Within-subject BMI measurements have greater consistency over time than do within-subject WFL measurements,^8^ suggesting greater stability of BMI compared with WFL. Existing guidelines suggest use of BMI for growth and obesity screening after 2 years of age.^3^ Applying the same metric for children younger than 2 years would therefore streamline clinical practice. Thus, if BMI replaced WFL for assessment of weight status in children younger than 2 years, it could improve monitoring of longitudinal growth patterns from infancy to adulthood without the need to transition between differing growth metrics after 2 years of age.

To date, few studies have examined the meaning of BMI calculated from recumbent length or the consequences of high BMI during infancy and early childhood. Because of these unanswered questions, BMI is not currently recommended for clinical use in children younger than 2 years.^3^ Recent studies, however, have indicated that BMI may be a suitable proxy of adiposity in older infants^35,36,37^ and may also provide information about future obesity^38,39,40^ and cardiometabolic risk^41,42,43^ during later childhood. Our findings also suggest that high BMI during the first 2 years of life is an indicator of adiposity and metabolic risk during early adolescence, with estimates that were comparable to those for high WFL. We acknowledge, however, that our findings would benefit from replication in other population cohorts from different settings. Furthermore, associations with outcomes other than adiposity or cardiometabolic risk might differ.

The strength of the associations observed was smaller for children in PROBIT than for those in Project Viva. We speculate that this difference is because children in PROBIT were larger during infancy than those in Project Viva (as indicated by the higher overweight prevalence in PROBIT infants than in Project Viva), whereas adolescent adiposity was higher in Project Viva than in PROBIT. It is likely that these growth metrics contribute a smaller explained variance for cardiometabolic risk markers, such as insulin resistance and metabolic risk score (as indicated by the small effect sizes in both cohorts), than true adiposity-related outcomes, such as FMI.

Our findings address important evidence gaps. First, we found that the CDC and WHO cut points for infant or early childhood overweight provided similar adjusted estimates and model estimations of cardiometabolic outcomes during early adolescence. This finding suggests that if pediatricians were to switch from using the CDC WFL 95th percentile or greater to WHO WFL or BMI 97.7th percentile or greater during the first 2 years of life, the ability to estimate future cardiometabolic outcomes would not be greatly affected. Second, we found that growth percentiles in children younger than 2 years that are indicative of potential health problems (ie, high WFL or BMI) were associated with direct measures of adiposity and cardiometabolic risk later in life. Third, we provided evidence of the clinical implications of using WFL or BMI percentiles in children younger than 2 years as indicators of future health outcomes beyond childhood. Previous studies^7,8^ were limited to associations with risk of obesity and to follow-up during childhood rather than adolescence.

Body mass index is a widely recommended metric for obesity screening in children.^44,45,46^ Children who were screened and underwent intensive behavioral interventions that encompassed nutritional counseling (eg, providing information about healthy eating, reading food labels, and encouraging the use of stimulus control) and physical activity had improvements in weight status for up to 12 months of age with minimal harm from screening.^46^ However, evidence favoring early life screening and subsequent interventions in children younger than 6 years remains scarce.^47^ Existing obesity prevention studies in early childhood have found only modest benefits, and few have examined whether there are benefits for later cardiometabolic health.^48^ Further research is needed to develop and test preventive interventions, especially for children who are diagnosed as overweight or obese during early life.

### Strengths and Limitations

Strengths of our study include its relatively large sample size of more than 13 000 children from 2 prospective cohorts, multiple measures of growth in early life, and a wide range of cardiometabolic outcomes during early adolescence obtained by highly trained research staff using standardized protocols. In addition, our study benefits from the variability in designs and populations in 2 different cohorts. The robustness and similarity of the findings in both cohorts despite differing confounding structures (degree of income inequality, health care systems) and different obesity prevalence suggest that bias attributable to uncontrolled (residual) confounding is an unlikely explanation for the observed associations.

Our study is not without limitations. First, we used both research-standard and routinely collected anthropometric measurements from well-child care visits across the first 2 years of life, which may be subject to differences in agreement.^49^ A recent study,^34^ however, identified high agreement between these 2 data sources when using the WFL or BMI to classify overweight status in children younger than 2 years. Second, the value of length-based indexes, such as the WFL or BMI, could be affected by inaccurate length assessments because of measurement difficulties in infants and toddlers, especially considering that length is squared when calculating the BMI. Third, we made no attempts to standardize the measurement of length during infancy across hospital or polyclinic sites in PROBIT because differences in length gain were not among the study’s major hypotheses during the first year of follow-up.^15^ Because we were unable to assess the reliability of infant length measurements in PROBIT, the associations of the WFL or BMI with early adolescent outcomes could have been attenuated by measurement error. Fourth, our study findings may not be generalizable to other racial/ethnic groups and populations because many of our participants were white (both cohorts) and university educated (in Project Viva). Fifth, some children were not followed up in both cohorts. In Project Viva, differences between children who were or were not followed up may limit the generalizability of our findings. In PROBIT, however, differences in characteristics between children followed up or not were small overall and therefore unlikely to have biased our findings. Sixth, the use of foot-to-foot bioimpedance methods in our study may underestimate adiposity compared with other methods, such as the 4-compartment model. Comparisons of the different methods, however, have reported the validity of bioimpedance to accurately rank individuals^16,50^ and groups.^51^ Seventh, we investigated multiple cardiometabolic outcomes, therefore increasing the risk of false-positive results. We chose not to adjust for multiple comparisons. Instead, the significance of our findings is based on the consistency of the associations observed across related outcomes.^52^ Eighth, our study addressed neither underweight status during the first 2 years of life using the WFL or BMI nor its associations with subsequent outcomes. Instead, our study focused on later cardiometabolic sequalae, which are more strongly associated with overweight than with underweight.^53^ We believe that the resolution of these limitations, however, would not alter our conclusions.

## Conclusions

We found that choice of the WFL vs BMI to define overweight during infancy and early childhood did not substantially affect associations with adiposity and cardiometabolic outcomes during early adolescence. Although our findings would benefit from replication in other population cohorts, they have implications for investigators seeking to use BMI as a growth metric for epidemiologic research and for practitioners monitoring the weight status of children younger than 2 years.

## Notes

Supplement. **eFigure.** Flowchart of Study Sample **eTable 1**. Intra-class Correlations (ICC) and Agreements Between CDC WFL, WHO WFL and WHO BMI z-Scores in Project Viva and PROBIT **eTable 2**. Characteristics of Subjects Included and Excluded From the Study in Project Viva and PROBIT **eTable 3**. Associations of Ever Overweight at 6-24 Months With Lean Mass, Adiposity and Metabolic Risk Score Components at Early Adolescence **eTable 4**. Associations of Being Overweight at 6 Months With Lean Mass, Adiposity and Metabolic Risk Score Components at Early Adolescence **eTable 5**. Associations of Being Overweight at 12 Months With Lean Mass, Adiposity and Metabolic Risk Score Components at Early Adolescence **eTable 6**. Associations of Being Overweight at 18 Months With Lean Mass, Adiposity and Metabolic Risk Score Components at Early Adolescence **eTable 7**. Associations of Being Overweight at 24 Months With Lean Mass, Adiposity and Metabolic Risk Score Components at Early Adolescence **eTable 8**. Associations of Number of Time Points Overweight at 6-24 Months With Lean Mass, Adiposity and Metabolic Risk Score Components at Early Adolescence
