# Supplementary material for: Association of Weight for Length vs Body Mass Index During the First 2 Years of Life With Cardiometabolic Risk in Early Adolescence
Source: JAMA Netw Open. 2018 Sep 21;1(5):e182460. doi: 10.1001/jamanetworkopen.2018.2460 (PMC6324504; doi:10.1001/jamanetworkopen.2018.2460)
Supplement: Supplement. — eFigure. Flowchart of Study Sample eTable 1. Intra-class Correlations (ICC) and Agreements Between CDC WFL, WHO WFL and WHO BMI z-Scores in Project Viva and PROBIT eTable 2. Characteristics of Subjects Included and Excluded From the Study in Project Viva and PROBIT eTable 3. Associations of Ever Overweight at 6-24 Months With Lean Mass, Adiposity and Metabolic Risk Score Components at Early Adolescence eTable 4. Associations of Being Overweight at 6 Months With Lean Mass, Adiposity and Metabolic Risk Score Components at Early Adolescence eTable 5. Associations of Being Overweight at 12 Months With Lean Mass, Adiposity and Metabolic Risk Score Components at Early Adolescence eTable 6. Associations of Being Overweight at 18 Months With Lean Mass, Adiposity and Metabolic Risk Score Components at Early Adolescence eTable 7. Associations of Being Overweight at 24 Months With Lean Mass, Adiposity and Metabolic Risk Score Components at Early Adolescence eTable 8. Associations of Number of Time Points Overweight at 6-24 Months With Lean Mass, Adiposity and Metabolic Risk Score Components at Early Adolescence [file jamanetwopen-1-e182460-s001.pdf]

Supplementary Online Content

Aris IM, Rifas-Shiman SL, Li L-J, et al. Association of Weight for Length vs Body Mass Index During the First 2 Years of Life With Cardiometabolic Risk in Early Adolescence. *JAMA Netw Open.* 2018;1(5):e182460. doi:10.1001/jamanetworkopen.2018.2460

eFigure. Flowchart of Study Sample

eTable 1. Intra-class Correlations (ICC) and Agreements Between CDC WFL, WHO WFL and WHO BMI z-Scores in Project Viva and PROBIT

eTable 2. Characteristics of Subjects Included and Excluded From the Study in Project Viva and PROBIT

eTable 3. Associations of Ever Overweight at 6-24 Months With Lean Mass, Adiposity and Metabolic Risk Score Components at Early Adolescence

eTable 4. Associations of Being Overweight at 6 Months With Lean Mass, Adiposity and Metabolic Risk Score Components at Early Adolescence

eTable 5. Associations of Being Overweight at 12 Months With Lean Mass, Adiposity and Metabolic Risk Score Components at Early Adolescence

eTable 6. Associations of Being Overweight at 18 Months With Lean Mass, Adiposity and Metabolic Risk Score Components at Early Adolescence

eTable 7. Associations of Being Overweight at 24 Months With Lean Mass, Adiposity and Metabolic Risk Score Components at Early Adolescence

eTable 8. Associations of Number of Time Points Overweight at 6-24 Months With Lean Mass, Adiposity and Metabolic Risk Score Components at Early Adolescence

This supplementary material has been provided by the authors to give readers additional information about their work.

© 2018 Aris IM et al. *JAMA Network Open.*

eFigure 1: Flow chart of study sample

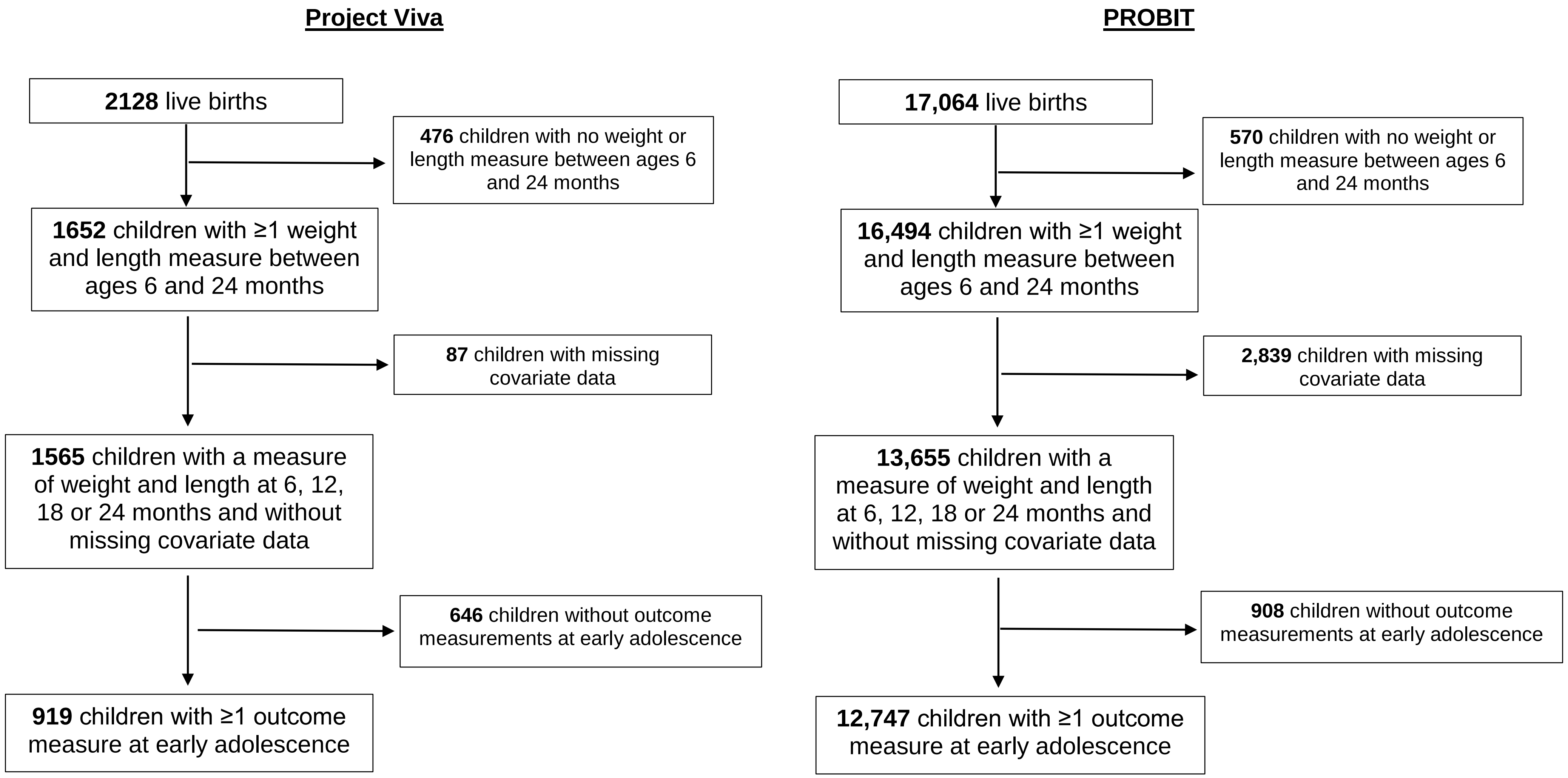

© 2018 Aris IM et al. *JAMA Network Open.*

eTable 1: Intra-class correlations (ICC) and agreements between CDC WFL, WHO WFL and WHO BMI z-scores in Project Viva and PROBIT

	Project Viva				PROBIT			
	**ICC for z-scores**	**% agreement for overweight cut-points**	**κ (95% CI) for overweight cut-points**	**p value for κ**	**ICC for z-scores**	**% agreement for overweight cut-points**	**κ (95% CI) for overweight cut-points**	**p value for κ**
**Month 6 (n=891)**					**Month 6 (n=12,594)**			
CDC vs WHO WFL	0.99	94.6	0.75 (0.69-0.82)	<0.01	0.99	95.9	0.77 (0.75-0.79)	<0.01
CDC vs WHO BMI	0.99	93.4	0.68 (0.61-0.76)	<0.01	0.99	95.0	0.72 (0.70-0.74)	<0.01
WHO WFL vs WHO BMI	0.99	98.8	0.93 (0.88-0.97)	<0.01	0.99	99.2	0.94 (0.93-0.95)	<0.01
**Month 12 (n=701)**					**Month 12 (n=12,707)**			
CDC vs WHO WFL	0.95	98.0	0.87 (0.80-0.94)	<0.01	0.95	96.6	0.89 (0.88-0.90)	<0.01
CDC vs WHO BMI	0.96	98.6	0.91 (0.85-0.96)	<0.01	0.96	96.1	0.87 (0.86-0.88)	<0.01
WHO WFL vs WHO BMI	0.98	98.0	0.86 (0.78-0.93)	<0.01	0.98	95.4	0.84 (0.83-0.85)	<0.01
**Month 18 (n=634)**					**Month 18 (n=2,965)**			
CDC vs WHO WFL	0.91	99.2	0.96 (0.92-0.99)	<0.01	0.93	99.1	0.96 (0.95-0.98)	<0.01
CDC vs WHO BMI	0.89	97.6	0.88 (0.81-0.94)	<0.01	0.91	96.6	0.87 (0.84-0.89)	<0.01
WHO WFL vs WHO BMI	0.98	97.2	0.85 (0.78-0.92)	<0.01	0.97	96.4	0.85 (0.83-0.88)	<0.01
**Month 24 (n=563)**					**Month 24 (n=4,590)**			
CDC vs WHO WFL	0.94	99.1	0.95 (0.90-0.99)	<0.01	0.95	98.7	0.94 (0.93-0.96)	<0.01
CDC vs WHO BMI	0.96	98.1	0.88 (0.80-0.95)	<0.01	0.94	96.3	0.84 (0.82-0.87)	<0.01
WHO WFL vs WHO BMI	0.98	98.9	0.93 (0.87-0.99)	<0.01	0.98	97.1	0.87 (0.85-0.89)	<0.01
**Ever overweight (n=919)**					**Ever overweight (n=12,747)**			
CDC vs WHO WFL	NA	94.5	0.84 (0.80-0.89)	<0.01	NA	94.9	0.87 (0.86-0.88)	<0.01
CDC vs WHO BMI	NA	93.6	0.80 (0.75-0.85)	<0.01	NA	93.3	0.83 (0.82-0.84)	<0.01
WHO WFL vs WHO BMI	NA	96.8	0.89 (0.85-0.93)	<0.01	NA	95.0	0.86 (0.85-0.87)	<0.01

NA: not applicable

© 2018 Aris IM et al. *JAMA Network Open.*

eTable 2: Characteristics of subjects included and excluded from the study in Project Viva and PROBIT

Project Viva	Included (n=919)	Excluded (n=1209)	p value
Maternal			
**Age**			0.02
<20 years	29 (3.2)	40 (3.3)	
20-34 years	610 (66.4)	867 (71.7)	
≥ 35 years	280 (30.5)	302 (25.0)	
**Education level**			<0.01
Not university educated	261 (28.4)	483 (40.8)	
University educated	658 (71.6)	702 (59.2)	
**Marital status**			0.09
Married/co-habituating	851 (92.6)	1072 (90.5)	
Not married	68 (7.4)	112 (9.5)	
**Maternal smoking history**			<0.01
Never smoked	650 (70.7)	793 (66.8)	
Smoked prior to pregnancy	184 (20.0)	214 (18.0)	
Smoked during pregnancy	85 (9.3)	181 (15.2)	
**Pre-pregnancy BMI (kg/m^2)**	24.8 (5.2)	24.9 (5.7)	0.58
**Total gestational weight gain (kg)**	15.6 (5.3)	15.5 (6.0)	0.84
**Glucose tolerance status**			0.72
Normoglycemia	764 (83.1)	942 (82.1)	
Isolated hyperglycemia	80 (8.7)	100 (8.7)	
Intermediate glucose intolerance	29 (3.2)	35 (3.1)	
Gestational diabetes	46 (5.0)	71 (6.2)	
**Hypertensive disorders of pregnancy**			0.15
Normal blood pressure	822 (89.4)	1023 (87.9)	
Gestational hypertension	63 (6.9)	73 (6.3)	
Chronic Hypertension	10 (1.1)	18 (1.6)	
Pre-eclampsia	24 (2.6)	50 (4.3)	
Child			
**Sex**			0.24
Male	460 (50.1)	636 (52.6)	
Female	459 (49.9)	573 (47.4)	
**Race/ethnicity**			<0.01
White	598 (65.1)	744 (62.5)	
Black	143 (15.6)	212 (17.8)	
Hispanic	39 (4.2)	89 (7.5)	
Asian	27 (2.9)	68 (5.7)	
Others	112 (12.2)	77 (6.5)	
**Gestational age at delivery (weeks)**	39.6 (1.6)	39.3 (2.2)	<0.01
**Birthweight-for-gestational age z-score (SD units)**	0.2 (1.0)	0.2 (1.0)	0.26
**Breastfeeding status at 6 months**			<0.01
Formula only	79 (9.1)	138 (15.8)	
Weaned	312 (35.9)	367 (42.1)	
Mixed feeding	239 (27.5)	178 (20.4)	
Breastmilk only	239 (27.5)	188 (21.6)	
**Ever overweight during 6–24 months**			0.44
No	758 (82.5)	615 (83.9)	
Yes	161 (17.5)	118 (16.1)	
**PROBIT**	**Included (n=12747)**	**Excluded (n=4299)**	**p value**
Maternal			
**Age**			<0.01
<20 years	1704 (13.6)	663 (16.0)	
20-34 years	10284 (82.1)	3315 (79.8)	
≥ 35 years	542 (4.3)	177 (4.3)	
**Education level**			<0.01
Did not complete university	11047 (86.7)	3683 (85.7)	
Completed university	1700 (13.3)	616 (14.3)	
**Marital status**			<0.01
Registered/unregistered marriage	12280 (96.3)	4071 (94.7)	
Unmarried	467 (3.7)	228 (5.3)	
**Smoking during pregnancy**			<0.01
No	12499 (98.1)	4158 (96.7)	
Yes	248 (1.9)	141 (3.3)	
**BMI at 6.5 years (kg/m^2)**	24.5 (4.4)	24.1 (4.2)	0.03
Child			
**Sex**			0.01
Male	6204 (48.7)	1951 (46.4)	
Female	6543 (51.3)	2254 (53.6)	
**Gestational age at delivery (weeks)**	39.4 (1.0)	39.4 (1.0)	0.44
**Birthweight-for-gestational age z-score (SD units)**	0.4 (1.0)	0.3 (0.9)	<0.01
**Ever overweight during 6–24 months**			<0.01
No	9622 (75.5)	2906 (77.6)	
Yes	3125 (24.5)	841 (22.4)	

© 2018 Aris IM et al. *JAMA Network Open.*

eTable 3: Associations of ever overweight at 6–24 months with lean mass, adiposity and metabolic risk score components at early adolescence

	Project Viva										PROBIT									
	N	CDC WFL [β (95% CI)]			WHO WFL [β (95% CI)]			WHO BMI [β (95% CI)]			N	CDC WFL [β (95% CI)]			WHO WFL [β (95% CI)]			WHO BMI [β (95% CI)]		
		Model 1[a]	Model 2[b]	F-statistic Model 2	Model 1[a]	Model 2[b]	F-statistic Model 2	Model 1[a]	Model 2[b]	F-statistic Model 2		Model 1[a]	Model 2[c]	F-statistic Model 2	Model 1[a]	Model 2[c]	F-statistic Model 2	Model 1[a]	Model 2[c]	F-statistic Model 2
**Lean mass**																				
Height z-score (SD units)	919	0.3 (0.1,0.4)	0.2 (0.01,0.3)	4.2	0.3 (0.1,0.4)	0.2 (0.02,0.4)	4.2	0.3 (0.1,0.4)	0.2 (0.0,0.3)	4.2	12530	0.08 (0.04,0.1)	0.03 (-0.01,0.07)	52.2	0.09 (0.05,0.1)	0.04 (0.0,0.8)	52.4	0.03 (-0.02,0.07)	-0.02 (-0.06,0.02)	52.1
**Overall adiposity**																				
BMI z-score (SD units)	916	0.6 (0.5,0.8)	0.4 (0.3,0.6)	11.8	0.7 (0.5,0.9)	0.5 (0.3,0.7)	11.9	0.7 (0.5,0.9)	0.5 (0.3,0.7)	12.0	12523	0.4 (0.3,0.4)	0.3 (0.3,0.4)	102.2	0.4 (0.4,0.5)	0.3 (0.3,0.4)	98.5	0.4 (0.3,0.4)	0.3 (0.3,0.4)	97.4
SS+TR (mm)	917	4.1 (2.0,6.2)	2.4 (0.4,4.4)	9.6	5.0 (2.7,7.4)	3.0 (0.8,5.2)	9.7	5.5 (3.2,7.9)	3.7 (1.6,5.9)	9.9	12523	2.6 (2.1,3.0)	2.7 (2.3,3.1)	65.6	2.6 (2.2,3.1)	2.9 (2.4,3.3)	65.5	2.6 (2.2,3.1)	2.7 (2.3,3.1)	64.3
Fat-mass index (kg/m^2)	912	1.4 (0.8,1.9)	0.9 (0.5,1.4)	17.1	1.6 (1.0,2.2)	1.1 (0.6,1.6)	17.3	1.8 (1.2,2.4)	1.4 (0.9,1.9)	17.8	12435	0.5 (0.4,0.6)	0.5 (0.4,0.6)	88.7	0.5 (0.4,0.6)	0.6 (0.5,0.7)	88.3	0.5 (0.4,0.6)	0.6 (0.5,0.7)	87.1
Obesity (Odds ratio)	916	2.4 (1.6,3.7)	1.9 (1.1,3.1)	153.9	2.6 (1.6,4.0)	2.0 (1.2,3.3)	154.2	3.1 (2.0,4.8)	2.6 (1.5,4.4)	160.2	12523	2.2 (1.9,2.6)	2.1 (1.8,2.5)	298.6	2.2 (1.9,2.6)	2.1 (1.8,2.5)	296.2	2.1 (1.8,2.5)	2.0 (1.7,2.4)	289.9
**Metabolic risk score components**																				
Waist circumference (cm)	919	5.5 (3.7,7.3)	3.9 (2.3,5.6)	11.3	6.4 (4.4,8.3)	4.5 (2.6,6.3)	11.4	7.0 (5.0,8.9)	5.4 (3.6,7.2)	11.9	12529	2.6 (2.3,2.9)	2.2 (1.9,2.5)	82.4	2.8 (2.5,3.1)	2.4 (2.0,2.7)	82.8	2.5 (2.2,2.8)	2.1 (1.8,2.4)	79.4
Systolic BP z-score (SD units)	911	0.09 (-0.05,0.2)	0.08 (-0.05,0.2)	1.2	0.06 (-0.08,0.2)	0.05 (-0.1,0.2)	1.1	0.03 (-0.1,0.2)	0.02 (-0.1,0.2)	1.1	12493	0.04 (0.01,0.08)	0.05 (0.02,0.08)	8.1	0.04 (0.01,0.07)	0.05 (0.01,0.08)	7.9	0.04 (0.01,0.07)	0.04 (0.01,0.08)	7.8
HOMA-IR	538	0.05 (-0.07,0.2)	0.05 (-0.07,0.17)	4.2	0.1 (-0.01,0.3)	0.09 (-0.03,0.22)	4.2	0.1 (-0.01,0.3)	0.1 (-0.03,0.23)	4.2	11771	0.02 (-0.02,0.06)	0.07 (0.03,0.1)	27.4	0.01 (-0.03,0.06)	0.08 (0.04,0.1)	27.5	0.01 (-0.03,0.06)	0.06 (0.01,0.1)	27.1
HDL-cholesterol (mg/dL)	608	-0.07 (-2.7,2.5)	-0.3 (-2.9,2.3)	2.1	-1.1 (-4.0,1.7)	-1.5 (-4.4,1.4)	2.2	-0.8 (-3.7,2.0)	-1.5 (-4.4,1.4)	2.2	NA	NA	NA	NA	NA	NA	NA	NA	NA	NA
Triglycerides (mg/dL)	607	-1.4 (-7.3,4.5)	0.7 (-5.4,6.8)	1.1	0.2 (-6.2,6.7)	2.6 (-4.1,9.2)	1.2	0.4 (-6.1,6.9)	2.5 (-4.2,9.2)	1.2	NA	NA	NA	NA	NA	NA	NA	NA	NA	NA
Apo A1 (g/L)	NA	NA	NA	NA	NA	NA	NA	NA	NA	NA	12046	0.0 (-0.01,0.02)	0.0 (-0.01,0.02)	2.1	0.0 (-0.01,0.02)	0.0 (-0.01,0.02)	2.1	0.0 (-0.01,0.02)	0.0 (-0.02,0.01)	2.1
**Global metabolic risk**																				
Metabolic risk score (units)	534	0.08 (-0.04,0.2)	0.08 (-0.04,0.2)	3.2	0.1 (0.0,0.3)	0.12 (-0.01,0.25)	3.2	0.1 (0.03,0.3)	0.12 (-0.01,0.26)	3.2	11442	0.09 (0.08,0.1)	0.09 (0.07,0.11)	32.2	0.1 (0.08,0.1)	0.09 (0.07,0.12)	32.2	0.09 (-0.07,0.1)	0.08 (0.06,0.1)	30.8

[a] Unadjusted

[b] Adjusted for maternal age, marital status, educational attainment, pre-pregnancy BMI, total gestational weight gain, smoking history, glucose tolerance status, and gestational hypertensive disorders and child gestational age at delivery, race/ethnicity, sex, birth weight for gestational age z-scores, breastfeeding status at 6 months and age at outcome measurement.

[c] Adjusted for maternal age, maternal BMI at 6.5 years, educational attainment, marital status, smoking during pregnancy, gestational age at delivery, child sex, birth weight for gestational age z-scores and age at outcome measurement.

© 2018 Aris IM et al. *JAMA Network Open.*

eTable 4: Associations of being overweight at 6 months with lean mass, adiposity and metabolic risk score components at early adolescence

	Project Viva										PROBIT									
	N	CDC WFL [β (95% CI)]			WHO WFL [β (95% CI)]			WHO BMI [β (95% CI)]			N	CDC WFL [β (95% CI)]			WHO WFL [β (95% CI)]			WHO BMI [β (95% CI)]		
		Model 1[a]	Model 2[b]	F-statistic Model 2	Model 1[a]	Model 2[b]	F-statistic Model 2	Model 1[a]	Model 2[b]	F-statistic Model 2		Model 1[a]	Model 2[c]	F-statistic Model 2	Model 1[a]	Model 2[c]	F-statistic Model 2	Model 1[a]	Model 2[c]	F-statistic Model 2
**Lean mass**																				
Height z-score (SD units)	891	0.04 (-0.15,0.22)	-0.01 (-0.19,0.17)	3.8	-0.01 (-0.2,0.2)	-0.09 (-0.3,0.1)	3.9	-0.01 (-0.2,0.2)	-0.08 (-0.3,0.2)	3.9	12379	-0.05 (-0.1,0.01)	-0.07 (-0.1,-0.02)	51.9	-0.06 (-0.1,0.01)	-0.09 (-0.1,-0.02)	51.9	-0.07 (-0.01,0.0)	-0.09 (-0.1,-0.02)	52.0
**Overall adiposity**																				
BMI z-score (SD units)	888	0.5 (0.3,0.7)	0.3 (0.1,0.5)	10.6	0.5 (0.3,0.7)	0.3 (0.1,0.5)	10.4	0.5 (0.2,0.7)	0.3 (0.1,0.6)	10.4	12373	0.3 (0.3,0.4)	0.3 (0.2,0.3)	82.6	0.3 (0.2,0.4)	0.2 (0.2,0.3)	78.4	0.3 (0.2,0.4)	0.3 (0.2,0.3)	78.3
SS+TR (mm)	889	1.9 (-0.7,4.5)	0.3 (-2.1,2.7)	9.3	1.0 (-2.1,4.1)	-0.4 (-3.3,2.4)	9.3	1.3 (-2.0,4.6)	0.2 (-2.8,3.2)	9.3	12372	1.7 (1.1,2.3)	1.7 (1.2,2.3)	55.8	1.6 (0.8,2.3)	1.5 (0.8,2.2)	54.6	1.6 (0.9,2.4)	1.6 (0.9,2.4)	54.7
Fat-mass index (kg/m^2)	884	0.5 (-0.1,1.2)	0.2 (-0.3,0.8)	15.8	0.5 (-0.3,1.2)	0.2 (-0.4,0.9)	15.8	0.5 (-0.3,1.3)	0.4 (-0.3,1.1)	15.8	12292	0.4 (0.3,0.5)	0.4 (0.3,0.5)	78.1	0.3 (0.2,0.5)	0.3 (0.2,0.5)	76.3	0.4 (0.2,0.5)	0.4 (0.2,0.5)	76.4
Obesity (Odds ratio)	888	1.4 (0.8,2.4)	0.9 (0.5,1.7)	146.3	1.2 (0.6,2.2)	0.8 (0.4,1.7)	146.6	1.4 (0.7,2.7)	1.0 (0.5,2.2)	146.2	12373	2.0 (1.6,2.4)	1.9 (1.5,2.3)	266.1	1.8 (1.4,2.3)	1.7 (1.3,2.2)	249.1	1.9 (1.5,2.5)	1.8 (1.4,2.3)	251.8
**Metabolic risk score components**																				
Waist circumference (cm)	892	3.7 (1.6,5.9)	2.0 (0.0,3.9)	10.0	3.9 (1.3,6.5)	2.1 (-0.3,4.5)	10.0	4.3 (1.5,7.0)	2.7 (0.1,5.2)	10.1	12378	1.7 (1.2,2.1)	1.4 (1.0,1.8)	69.2	1.6 (1.1,2.1)	1.2 (0.7,1.7)	67.5	1.6 (1.1,2.1)	1.2 (0.7,1.7)	67.5
Systolic BP z-score (SD units)	884	0.08 (-0.08,0.2)	0.05 (-0.1,0.2)	1.3	0.02 (-0.2,0.2)	-0.01 (-0.2,0.2)	1.3	0.04 (-0.2,0.2)	0.01 (-0.2,0.2)	1.3	12342	0.07 (0.03,0.1)	0.07 (0.03,0.1)	8.2	0.06 (0.01,0.1)	0.05 (0.0,0.1)	7.7	0.07 (0.01,0.1)	0.06 (0.0,0.1)	7.8
HOMA-IR	521	-0.03 (-0.2,0.1)	-0.02 (-0.1,0.1)	4.2	-0.04 (-0.2,0.1)	-0.03 (-0.2,0.1)	4.2	-0.08 (-0.3,0.1)	-0.06 (-0.2,0.1)	4.2	11634	0.01 (-0.05,0.07)	0.04 (-0.01,0.1)	26.1	-0.01 (-0.08,0.06)	0.03 (-0.04,0.1)	26.0	-0.03 (-0.1,0.04)	0.01 (-0.06,0.08)	26.0
HDL-cholesterol (mg/dL)	590	0.2 (-2.9,3.2)	0.2 (-2.8,3.3)	2.0	0.7 (-3.0,4.3)	0.8 (-2.9,4.5)	2.0	0.2 (-3.7,4.1)	-0.1 (-4.0,3.8)	2.0	NA	NA	NA	NA	NA	NA	NA	NA	NA	NA
Triglycerides (mg/dL)	589	-1.0 (-7.9,5.9)	0.5 (-6.6,7.6)	1.2	-2.9 (-11.3,5.4)	-1.4 (-9.9,7.1)	1.2	-6.0 (-14.9,2.9)	-4.0 (-13.1,5.0)	1.3	NA	NA	NA	NA	NA	NA	NA	NA	NA	NA
Apo A1 (g/L)	NA	NA	NA	NA	NA	NA	NA	NA	NA	NA	11904	-0.01 (-0.03,0.01)	-0.01 (-0.03,0.01)	2.3	-0.01 (-0.03,0.02)	-0.01 (-0.04,0.01)	2.3	-0.02 (-0.04,0.01)	-0.02 (-0.05,0.0)	2.4
**Global metabolic risk**																				
Metabolic risk score (units)	518	0.0 (-0.1,0.1)	-0.01 (-0.2,0.1)	3.1	-0.04 (-0.2,0.1)	-0.07 (-0.2,0.1)	3.1	-0.06 (-0.2,0.1)	-0.07 (-0.2,0.1)	3.1	11308	0.06 (0.03,0.08)	0.05 (0.02,0.08)	27.9	0.05 (0.02,0.09)	0.04 (0.0,0.07)	27.5	0.05 (0.01,0.08)	0.04 (0.0,0.07)	27.4

[a] Unadjusted
[b] Adjusted for maternal age, marital status, educational attainment, pre-pregnancy BMI, total gestational weight gain, smoking history, glucose tolerance status, and gestational hypertensive disorders and child gestational age at delivery, race/ethnicity, sex, birth weight for gestational age z-scores, breastfeeding status at 6 months and age at outcome measurement.
[c] Adjusted for maternal age, maternal BMI at 6.5 years, educational attainment, marital status, smoking during pregnancy, gestational age at delivery, child sex, birth weight for gestational age z-scores and age at outcome measurement.

© 2018 Aris IM et al. *JAMA Network Open.*

eTable 5: Associations of being overweight at 12 months with lean mass, adiposity and metabolic risk score components at early adolescence

	Project Viva										PROBIT									
	N	CDC WFL [β (95% CI)]			WHO WFL [β (95% CI)]			WHO BMI [β (95% CI)]			N	CDC WFL [β (95% CI)]			WHO WFL [β (95% CI)]			WHO BMI [β (95% CI)]		
		Model 1[a]	Model 2[b]	F-statistic Model 2	Model 1[a]	Model 2[b]	F-statistic Model 2	Model 1[a]	Model 2[b]	F-statistic Model 2		Model 1[a]	Model 2[c]	F-statistic Model 2	Model 1[a]	Model 2[c]	F-statistic Model 2	Model 1[a]	Model 2[c]	F-statistic Model 2
**Lean mass**																				
Height z-score (SD units)	701	0.3 (0.1,0.6)	0.2 (-0.05,0.5)	3.3	0.4 (0.1,0.7)	0.2 (-0.1,0.5)	3.3	0.3 (-0.02,0.5)	0.1 (-0.1,0.4)	3.2	12491	0.1 (0.07,0.2)	0.05 (0.0,0.1)	52.4	0.1 (0.1,0.2)	0.08 (0.03,0.1)	52.9	0.04 (-0.01,0.09)	0.0 (-0.05,0.04)	51.9
**Overall adiposity**																				
BMI z-score (SD units)	699	0.5 (0.2,0.8)	0.3 (0.1,0.6)	7.4	0.6 (0.3,0.9)	0.4 (0.1,0.7)	7.4	0.6 (0.3,0.8)	0.4 (0.2,0.7)	7.5	12485	0.4 (0.4,0.5)	0.3 (0.3,0.4)	95.4	0.4 (0.4,0.5)	0.4 (0.3,0.4)	94.3	0.4 (0.3,0.4)	0.3 (0.3,0.4)	91.6
SS+TR (mm)	700	2.8 (-0.8,6.4)	1.9 (-1.5,5.3)	6.3	4.7 (0.7,8.7)	3.1 (-0.7,6.9)	6.3	4.3 (0.4,8.2)	3.7 (0.03,7.3)	6.4	12484	2.7 (2.2,3.1)	2.9 (2.4,3.3)	64.0	2.9 (2.4,3.4)	3.3 (2.8,3.8)	65.4	2.6 (2.1,3.2)	2.8 (2.3,3.3)	62.1
Fat-mass index (kg/m^2)	699	1.3 (0.4,2.2)	1.0 (0.2,1.7)	12.0	1.8 (0.8,2.8)	1.1 (0.3,2.0)	12.0	1.6 (0.6,2.6)	1.3 (0.4,2.1)	12.2	12397	0.5 (0.4,0.6)	0.5 (0.4,0.6)	84.8	0.5 (0.4,0.6)	0.6 (0.5,0.7)	85.5	0.5 (0.4,0.6)	0.5 (0.4,0.6)	83.6
Obesity (Odds ratio)	699	2.4 (1.3,4.5)	1.9 (0.8,4.2)	120.5	2.8 (1.5,5.5)	2.1 (0.9,4.9)	121.0	2.8 (1.5,5.4)	2.7 (1.2,6.6)	123.3	12485	2.0 (1.7,2.4)	1.9 (1.6,2.3)	278.3	2.2 (1.8,2.6)	2.1 (1.7,2.5)	284.0	2.0 (1.7,2.5)	2.0 (1.6,2.4)	277.3
**Metabolic risk score components**																				
Waist circumference (cm)	702	4.9 (1.9,8.0)	3.5 (0.7,6.3)	7.3	5.9 (2.5,9.3)	3.9 (0.8,7.1)	7.3	5.4 (2.2,8.7)	4.4 (1.4,7.4)	7.4	12490	2.7 (2.3,3.0)	2.2 (1.9,2.6)	79.7	3.1 (2.7,3.4)	2.6 (2.3,3.0)	82.2	2.5 (2.2,2.9)	2.1 (1.8,2.5)	77.0
Systolic BP z-score (SD units)	696	0.1 (-0.1,0.4)	0.1 (-0.1,0.4)	1.3	0.1 (-0.1,0.4)	0.1 (-0.1,0.4)	1.3	0.1 (-0.1,0.4)	0.1 (-0.1,0.4)	1.3	12454	0.05 (0.01,0.08)	0.05 (0.02,0.09)	8.0	0.05 (0.01,0.08)	0.05 (0.01,0.09)	7.9	0.05 (0.02,0.09)	0.06 (0.02,0.1)	8.0
HOMA-IR	408	0.0 (-0.2,0.2)	-0.02 (-0.2,0.2)	2.4	0.04 (-0.2,0.3)	0.0 (-0.2,0.2)	2.4	0.01 (-0.2,0.2)	0.01 (-0.2,0.2)	2.4	11734	0.02 (-0.03,0.06)	0.08 (0.03,0.13)	27.3	0.01 (-0.05,0.06)	0.09 (0.04,0.14)	27.4	0.01 (-0.04,0.06)	0.06 (0.01,0.11)	27.0
HDL-cholesterol (mg/dL)	463	0.7 (-3.5,4.9)	-0.3 (-4.6,3.9)	1.9	-1.7 (-6.2,2.8)	-2.8 (-7.4,1.8)	1.9	0.9 (-3.6,5.5)	-0.2 (-4.8,4.4)	1.9	NA	NA	NA	NA	NA	NA	NA	NA	NA	NA
Triglycerides (mg/dL)	462	-3.2 (-13.2,6.8)	-0.8 (-11.2,9.6)	1.0	0.2 (-10.5,10.9)	2.8 (-8.4,13.9)	1.1	-0.3 (-10.5,11.2)	2.7 (-8.6,13.9)	1.1	NA	NA	NA	NA	NA	NA	NA	NA	NA	NA
Apo A1 (g/L)	NA	NA	NA	NA	NA	NA	NA	NA	NA	NA	12008	0.01 (-0.01,0.03)	0.0 (-0.01,0.02)	2.1	0.01 (-0.01,0.03)	0.0 (-0.01,0.02)	2.1	0.0 (-0.01,0.02)	0.0 (-0.02,0.01)	2.1
**Global metabolic risk**																				
Metabolic risk score (units)	405	0.03 (-0.2,0.2)	0.05 (-0.2,0.3)	2.3	0.1 (-0.1,0.3)	0.1 (-0.1,0.3)	2.3	0.05 (-0.2,0.3)	0.07 (-0.2,0.3)	2.3	11407	0.1 (0.08,0.1)	0.1 (0.07,0.12)	32.1	0.1 (0.09,0.1)	0.11 (0.08,0.14)	32.5	0.09 (0.07,0.1)	0.08 (0.06,0.11)	30.4

[a] Unadjusted
[b] Adjusted for maternal age, marital status, educational attainment, pre-pregnancy BMI, total gestational weight gain, smoking history, glucose tolerance status, and gestational hypertensive disorders and child gestational age at delivery, race/ethnicity, sex, birth weight for gestational age z-scores, breastfeeding status at 6 months and age at outcome measurement.
[c] Adjusted for maternal age, maternal BMI at 6.5 years, educational attainment, marital status, smoking during pregnancy, gestational age at delivery, child sex, birth weight for gestational age z-scores and age at outcome measurement.

© 2018 Aris IM et al. *JAMA Network Open.*

eTable 6: Associations of being overweight at 18 months with lean mass, adiposity and metabolic risk score components at early adolescence

	Project Viva										PROBIT									
	N	CDC WFL [β (95% CI)]			WHO WFL [β (95% CI)]			WHO BMI [β (95% CI)]			N	CDC WFL [β (95% CI)]			WHO WFL [β (95% CI)]			WHO BMI [β (95% CI)]		
		**Model 1[a]**	**Model 2[b]**	**F-statistic Model 2**	**Model 1[a]**	**Model 2[b]**	**F-statistic Model 2**	**Model 1[a]**	**Model 2[b]**	**F-statistic Model 2**		**Model 1[a]**	**Model 2[c]**	**F-statistic Model 2**	**Model 1[a]**	**Model 2[c]**	**F-statistic Model 2**	**Model 1[a]**	**Model 2[c]**	**F-statistic Model 2**
**Lean mass**																				
Height z-score (SD units)	634	0.5 (0.2,0.7)	0.3 (0.1,0.6)	3.2	0.5 (0.2,0.7)	0.3 (0.1,0.6)	3.2	0.3 (0.1,0.6)	0.2 (-0.03,0.5)	3.1	2908	0.2 (0.1,0.3)	0.1 (0.03,0.2)	12.1	0.2 (0.1,0.3)	0.1 (0.04,0.2)	12.2	0.1 (0.05,0.2)	0.1 (0.0,0.2)	11.9
**Overall adiposity**																				
BMI z-score (SD units)	634	0.9 (0.7,1.2)	0.8 (0.5,1.0)	8.7	0.9 (0.6,1.2)	0.7 (0.5,1.0)	8.5	0.9 (0.6,1.1)	0.7 (0.5,1.0)	8.5	2906	0.6 (0.5,0.7)	0.5 (0.4,0.6)	25.9	0.6 (0.5,0.7)	0.5 (0.4,0.6)	25.4	0.6 (0.5,0.7)	0.5 (0.4,0.6)	25.4
SS+TR (mm)	632	8.8 (5.3,12.2)	7.1 (3.8,10.4)	7.7	7.6 (4.0,11.3)	5.9 (2.9,9.3)	7.4	7.5 (4.0,11.0)	6.0 (2.7,9.3)	7.4	2907	4.5 (3.3,5.7)	4.5 (3.4,5.7)	15.5	4.5 (3.3,5.7)	4.4 (3.2,5.6)	15.0	4.2 (3.1,5.3)	4.1 (3.0,5.2)	14.9
Fat-mass index (kg/m^2)	633	2.7 (1.9,3.6)	2.2 (1.5,3.0)	13.2	2.6 (1.7,3.4)	2.1 (1.3,2.8)	12.8	2.5 (1.6,3.4)	2.1 (1.4,2.9)	13.0	2895	1.0 (0.8,1.3)	1.1 (0.8,1.3)	25.3	1.0 (0.8,1.3)	1.1 (0.8,1.3)	24.8	1.0 (0.8,1.2)	1.0 (0.8,1.2)	24.5
Obesity (Odds ratio)	634	4.6 (2.6,8.1)	5.0 (2.4,10.1)	126.3	3.6 (2.0,6.6)	3.7 (1.7,7.7)	118.7	4.3 (2.4,7.6)	4.6 (2.2,9.5)	124.0	2906	3.2 (2.3,4.5)	2.9 (2.0,4.1)	101.9	3.3 (2.3,4.7)	2.9 (2.0,4.1)	101.4	3.0 (2.1,4.2)	2.7 (1.9,3.9)	99.5
**Metabolic risk score components**																				
Waist circumference (cm)	635	9.9 (7.0,12.7)	8.0 (5.0,10.7)	9.0	9.1 (6.1,12.0)	7.3 (4.5,10.1)	8.6	9.3 (6.4, 12.2)	7.6 (4.9,10.3)	8.8	2909	4.6 (3.7,5.4)	4.1 (3.3,4.9)	23.8	4.7 (3.8,5.5)	4.1 (3.3,5.0)	23.7	4.3 (3.5,5.1)	3.9 (3.1,4.7)	23.6
Systolic BP z-score (SD units)	630	0.02 (-0.2,0.2)	0.0 (-0.2,0.2)	1.2	0.01 (-0.2,0.2)	-0.02 (-0.2,0.2)	1.2	-0.04 (-0.3,0.2)	-0.07 (-0.3,0.1)	1.2	2899	0.08 (0.0,0.16)	0.08 (-0.01,0.16)	3.1	0.07 (-0.02,0.16)	0.06 (-0.02,0.15)	3.0	0.05 (-0.03,0.13)	0.05 (-0.04,0.13)	2.9
HOMA-IR	374	0.3 (0.1,0.5)	0.3 (0.1,0.5)	3.0	0.3 (0.1,0.5)	0.3 (0.1,0.4)	2.9	0.3 (0.1,0.5)	0.3 (0.1,0.4)	2.9	2756	0.04 (-0.07,0.1)	0.09 (-0.01,0.2)	6.5	0.03 (-0.08,0.1)	0.09 (-0.02,0.2)	6.5	0.06 (-0.05,0.1)	0.09 (-0.01,0.2)	6.6
HDL-cholesterol (mg/dL)	420	-3.5 (-7.7,0.7)	-3.7 (-8.0,0.6)	1.9	-3.1 (-7.4,1.2)	-3.3 (-7.7,1.1)	1.9	-4.6 (-8.9,-0.4)	-5.1 (-9.5,-0.7)	2.0	NA	NA	NA	NA	NA	NA	NA	NA	NA	NA
Triglycerides (mg/dL)	419	5.2 (-4.8,15.1)	6.5 (-3.9,16.8)	1.4	5.8 (-4.5,16.0)	6.8 (-3.8,17.4)	1.4	5.0 (-5.1,15.2)	6.7 (-4.0,17.3)	1.4	NA	NA	NA	NA	NA	NA	NA	NA	NA	NA
Apo A1 (g/L)	NA	NA	NA	NA	NA	NA	NA	NA	NA	NA	2798	0.0 (-0.04,0.04)	0.0 (-0.04,0.04)	1.2	0.01 (-0.03,0.05)	0.0 (-0.04,0.04)	1.2	0.01 (-0.02,0.05)	0.01 (-0.02,0.05)	1.2
**Global metabolic risk**																				
Metabolic risk score (units)	373	0.3 (0.2,0.5)	0.3 (0.1,0.5)	2.7	0.3 (0.1,0.5)	0.3 (0.1,0.5)	2.6	0.3 (0.1,0.5)	0.3 (0.1,0.5)	2.6	2690	0.2 (0.1,0.2)	0.14 (0.09,0.19)	10.4	0.2 (0.1,0.2)	0.15 (0.09,0.21)	10.6	0.2 (0.1,0.2)	0.15 (0.09,0.20)	10.7

[a] Unadjusted
[b] Adjusted for maternal age, marital status, educational attainment, pre-pregnancy BMI, total gestational weight gain, smoking history, glucose tolerance status, and gestational hypertensive disorders and child gestational age at delivery, race/ethnicity, sex, birth weight for gestational age z-scores, breastfeeding status at 6 months and age at outcome measurement.
[c] Adjusted for maternal age, maternal BMI at 6.5 years, educational attainment, marital status, smoking during pregnancy, gestational age at delivery, child sex, birth weight for gestational age z-scores and age at outcome measurement.

© 2018 Aris IM et al. *JAMA Network Open.*

eTable 7: Associations of being overweight at 24 months with lean mass, adiposity and metabolic risk score components at early adolescence

	Project Viva										PROBIT									
	N	CDC WFL [β (95% CI)]			WHO WFL [β (95% CI)]			WHO BMI [β (95% CI)]			N	CDC WFL [β (95% CI)]			WHO WFL [β (95% CI)]			WHO BMI [β (95% CI)]		
		Model 1[a]	Model 2[b]	F-statistic Model 2	Model 1[a]	Model 2[b]	F-statistic Model 2	Model 1[a]	Model 2[b]	F-statistic Model 2		Model 1[a]	Model 2[c]	F-statistic Model 2	Model 1[a]	Model 2[c]	F-statistic Model 2	Model 1[a]	Model 2[c]	F-statistic Model 2
**Lean mass**																				
Height z-score (SD units)	563	0.5 (0.2,0.7)	0.3 (0.0,0.6)	2.9	0.5 (0.2,0.8)	0.3 (0.0,0.6)	3.0	0.5 (0.2,0.8)	0.3 (-0.01,0.6)	2.9	4503	0.1 (0.05,0.2)	0.09 (0.01,0.2)	18.3	0.1 (0.0,0.2)	0.05 (-0.03,0.1)	18.1	0.03 (-0.06,0.1)	-0.01 (-0.1,0.07)	18.0
**Overall adiposity**																				
BMI z-score (SD units)	563	1.0 (0.7,1.3)	0.8 (0.5,1.0)	6.9	1.0 (0.6,1.3)	0.7 (0.4,1.0)	6.7	1.0 (0.7,1.3)	0.8 (0.5,1.1)	6.9	4502	0.6 (0.5,0.6)	0.5 (0.4,0.6)	42.8	0.6 (0.5,0.7)	0.5 (0.4,0.6)	42.1	0.5 (0.4,0.6)	0.4 (0.3,0.5)	40.2
SS+TR (mm)	563	10.8 (7.2,14.5)	9.0 (5.5,12.6)	5.8	10.4 (6.6,14.3)	8.5 (4.8,12.3)	5.6	11.7 (7.7,15.6)	9.4 (5.5,13.2)	5.7	4501	4.1 (3.1,5.0)	4.2 (3.3,5.1)	29.8	3.9 (2.9,4.9)	4.0 (3.1,5.0)	28.7	3.6 (2.7,4.5)	3.5 (2.6,4.4)	27.9
Fat-mass index (kg/m^2)	562	2.9 (2.0,3.8)	2.2 (1.4,3.0)	10.1	2.9 (1.9,3.8)	2.2 (1.4,3.0)	9.9	3.2 (2.3,4.2)	2.4 (1.5,3.3)	10.1	4483	0.9 (0.7,1.1)	0.9 (0.7,1.1)	38.3	0.9 (0.7,1.1)	0.9 (0.7,1.1)	37.8	0.8 (0.6,1.0)	0.8 (0.6,1.0)	36.0
Obesity (Odds ratio)	563	4.2 (2.2,8.0)	3.8 (1.7,8.8)	91.7	4.3 (2.2,8.5)	4.1 (1.7,9.6)	91.9	5.6 (2.8,11.1)	5.2 (2.2,12.3)	95.5	4502	2.7 (2.0,3.6)	2.4 (1.8,3.3)	149.3	2.6 (1.9,3.5)	2.3 (1.7,3.2)	146.4	2.3 (1.7,3.1)	2.0 (1.5,2.8)	140.3
**Metabolic risk score components**																				
Waist circumference (cm)	564	9.7 (6.7,12.7)	7.9 (4.9,10.8)	6.3	9.8 (6.6,12.9)	7.9 (4.9,11.0)	6.2	10.6 (7.4,13.9)	8.7 (5.6,11.9)	6.3	4503	3.8 (3.1,4.5)	3.3 (2.7,4.0)	31.9	3.7 (3.0,4.4)	3.2 (2.5,3.9)	30.9	3.1 (2.5,3.8)	2.6 (2.0,3.2)	29.1
Systolic BP z-score (SD units)	557	0.09 (-0.1,0.3)	0.08 (-0.2,0.3)	1.3	0.09 (-0.2,0.3)	0.07 (-0.2,0.3)	1.3	0.1 (-0.1,0.4)	0.1 (-0.1,0.4)	1.3	4493	0.02 (-0.05,0.08)	0.02 (-0.05,0.09)	4.6	0.02 (-0.05,0.09)	0.03 (-0.05,0.1)	4.6	0.04 (-0.03,0.1)	0.04 (-0.03,0.1)	4.7
HOMA-IR	338	0.3 (0.1,0.5)	0.3 (0.1,0.5)	2.9	0.3 (0.1,0.5)	0.3 (0.1,0.5)	2.8	0.3 (0.0,0.5)	0.2 (-0.02,0.5)	2.6	4242	0.08 (-0.01,0.2)	0.13 (0.04,0.22)	9.5	0.1 (0.0,0.2)	0.14 (0.05,0.24)	9.6	0.1 (0.01,0.2)	0.13 (0.04,0.22)	9.5
HDL-cholesterol (mg/dL)	378	-2.4 (-6.9,2.2)	-4.1 (-8.7,0.6)	2.1	-1.7 (-6.5,3.0)	-3.2 (-8.1,1.7)	2.0	-1.5 (-6.5,3.6)	-4.0 (-9.2,1.3)	2.1	NA	NA	NA	NA	NA	NA	NA	NA	NA	NA
Triglycerides (mg/dL)	377	0.9 (-9.6,11.4)	3.1 (-8.1,14.3)	1.0	-1.4 (-12.5,9.7)	-0.7 (-12.4,11.1)	1.0	-5.2 (-16.9,6.5)	-4.6 (-17.1,8.0)	1.1	NA	NA	NA	NA	NA	NA	NA	NA	NA	NA
Apo A1 (g/L)	NA	NA	NA	NA	NA	NA	NA	NA	NA	NA	4339	0.0 (-0.03,0.04)	0.0 (-0.03,0.03)	1.3	0.0 (-0.03,0.04)	0.0 (-0.03,0.03)	1.3	0.0 (-0.03,0.03)	0.0 (-0.03,0.03)	1.3
**Global metabolic risk**																				
Metabolic risk score (units)	335	0.3 (0.1,0.5)	0.3 (0.1,0.5)	2.9	0.3 (0.04,0.5)	0.3 (0.04,0.5)	2.8	0.3 (0.01,0.5)	0.3 (0.0,0.5)	2.7	4121	0.1 (0.08,0.2)	0.11 (0.06,0.15)	12.3	0.1 (0.07,0.2)	0.1 (0.06,0.15)	12.1	0.1 (0.06,0.2)	0.09 (0.04,0.13)	11.8

[a] Unadjusted
[b] Adjusted for maternal age, marital status, educational attainment, pre-pregnancy BMI, total gestational weight gain, smoking history, glucose tolerance status, and gestational hypertensive disorders and child gestational age at delivery, race/ethnicity, sex, birth weight for gestational age z-scores, breastfeeding status at 6 months and age at outcome measurement.
[c] Adjusted for maternal age, maternal BMI at 6.5 years, educational attainment, marital status, smoking during pregnancy, gestational age at delivery, child sex, birth weight for gestational age z-scores and age at outcome measurement.

© 2018 Aris IM et al. *JAMA Network Open.*

eTable 8: Associations of number of timepoints overweight at 6–24 months with lean mass, adiposity and metabolic risk score components at early adolescence

	**Lean mass**		**Total adiposity**								**Metabolic risk score components**						**MetS risk score**	
	**Height z-score**		**BMI z-score**		**SS+TR**		**Fat mass index**		**Obesity**		**Waist Circumference**		**Systolic BP z-score**		**HOMA-IR**			
	**Viva[a]**	**PROBIT[b]**	**Viva[a]**	**PROBIT[b]**	**Viva[a]**	**PROBIT[b]**	**Viva[a]**	**PROBIT[b]**	**Viva[a]**	**PROBIT[b]**	**Viva[a]**	**PROBIT[b]**	**Viva[a]**	**PROBIT[b]**	**Viva[a]**	**PROBIT[b]**	**Viva[a]**	**PROBIT[b]**
**CDC WFL**																		
0	ref	ref	ref	ref	ref	ref	ref	ref	ref	ref	ref	ref	ref	ref	ref	ref	ref	ref
1	0.1 (-0.06,0.3)	0.03 (-0.02,0.07)	0.3 (0.1,0.5)	0.3 (0.2,0.3)	0.6 (-1.8,2.9)	2.1 (1.6,2.6)	0.4 (-0.1,0.9)	0.4 (0.3,0.5)	1.1 (0.6,2.2)	1.7 (1.4,2.1)	2.2 (0.3,4.1)	1.7 (1.4,2.1)	0.03 (-0.1,0.2)	0.03 (0.0,0.07)	-0.06 (-0.2,0.1)	0.05 (0.0,0.1)	-0.03 (-0.2,0.1)	0.07 (0.04,0.09)
2	0.3 (-0.05,0.6)	0.03 (-0.04,0.09)	0.7 (0.4,1.0)	0.5 (0.4,0.5)	6.7 (2.6,10.7)	3.6 (2.9,4.3)	2.2 (1.2,3.1)	0.7 (0.6,0.9)	5.2 (2.3,11.7)	2.7 (2.1,3.4)	8.5 (5.1,11.8)	2.7 (2.2,3.2)	0.3 (0.1,0.6)	0.09 (0.03,0.14)	0.4 (0.1,0.6)	0.1 (0.04,0.2)	0.3 (0.1,0.6)	0.13 (0.09,0.17)
3–4	0.2 (-0.2,0.6)	0.06 (-0.07,0.2)	0.7 (0.4,1.1)	0.7 (0.6,0.8)	5.4 (0.9,9.9)	5.8 (4.4,7.3)	1.7 (0.7,2.7)	1.3 (1.1,1.6)	2.1 (0.7,6.4)	3.7 (2.5,5.6)	5.8 (2.0,9.5)	4.9 (3.9,5.9)	-0.04 (-0.4,0.3)	0.09 (-0.2,0.2)	0.1 (-0.1,0.4)	0.2 (0.02,0.3)	0.2 (-0.03,0.5)	0.14 (0.07,0.21)
**WHO WFL**																		
0	ref	ref	ref	ref	ref	ref	ref	ref	ref	ref	ref	ref	ref	ref	ref	ref	ref	ref
1	0.2 (-0.02,0.4)	0.03 (-0.02,0.08)	0.4 (0.2,0.6)	0.3 (0.2,0.3)	1.8 (-0.8,4.4)	2.3 (1.8,2.8)	0.7 (0.1,1.3)	0.5 (0.4,0.6)	1.6 (0.8,3.0)	1.8 (1.5,2.2)	3.1 (1.0,5.3)	1.9 (1.6,2.3)	0.03 (-0.2,0.2)	0.04 (0.0,0.08)	0.02 (-0.1,0.2)	0.06 (0.01,0.1)	0.03 (-0.1,0.2)	0.07 (0.05,0.1)
2	0.3 (-0.07,0.7)	0.06 (-0.01,0.1)	0.7 (0.3,1.0)	0.5 (0.4,0.5)	4.8 (0.0,9.5)	3.9 (3.1,4.7)	1.9 (0.8,3.0)	0.7 (0.6,0.9)	3.2 (1.1,9.1)	2.6 (2.0,3.4)	8.0 (4.0,11.9)	2.9 (2.3,3.5)	0.2 (-0.1,0.5)	0.05 (-0.01,0.11)	0.4 (0.1,0.7)	0.1 (0.04,0.2)	0.4 (0.1,0.7)	0.12 (0.08,0.16)
3–4	0.1 (-0.3,0.5)	0.06 (-0.09,0.2)	0.8 (0.4,1.1)	0.8 (0.6,0.9)	5.9 (1.0,10.8)	6.6 (4.8,8.3)	2.0 (0.9,3.1)	1.6 (1.2,1.9)	2.8 (0.9,8.4)	4.0 (2.5,6.4)	6.2 (2.2,10.3)	5.8 (4.6,7.0)	0.01 (-0.3,0.3)	0.1 (0.0,0.3)	0.07 (-0.2,0.4)	0.2 (0.04,0.4)	0.1 (-0.1,0.4)	0.2 (0.11,0.28)
**WHO BMI**																		
0	ref	ref	ref	ref	ref	ref	ref	ref	ref	ref	ref	ref	ref	ref	ref	ref	ref	ref
1	0.2 (-0.04,0.4)	-0.02 (-0.07,0.02)	0.4 (0.2,0.6)	0.3 (0.2,0.3)	2.5 (-0.1,5.1)	2.2 (1.7,2.7)	1.0 (0.4,1.6)	0.4 (0.3,0.5)	1.9 (1.0,3.7)	1.7 (1.4,2.1)	4.4 (2.3,6.6)	1.7 (1.4,2.1)	-0.03 (-0.2,0.1)	0.03 (-0.01,0.07)	0.05 (-0.1,0.2)	0.04 (-0.01,0.09)	0.06 (-0.1,0.2)	0.06 (0.04,0.09)
2	0.4 (0.03,0.7)	-0.01 (-0.08,0.07)	0.7 (0.4,1.0)	0.5 (0.4,0.5)	6.4 (2.2,10.6)	3.6 (2.7,4.4)	2.0 (1.0,2.9)	0.7 (0.6,0.9)	4.2 (1.8,10.2)	2.7 (2.1,3.5)	7.4 (3.9,10.9)	2.7 (2.1,3.3)	0.09 (-0.2,0.4)	0.07 (0.01,0.13)	0.3 (0.03,0.5)	0.08 (0.0,0.2)	0.3 (0.07,0.6)	0.11 (0.07,0.15)
3–4	-0.1 (-0.6,0.3)	-0.04 (-0.2,0.1)	0.8 (0.4,1.2)	0.7 (0.5,0.9)	5.8 (0.1,11.5)	5.8 (4.1,7.6)	2.1 (0.8,3.4)	1.4 (1.1,1.7)	3.7 (1.1,13.1)	3.8 (2.3,6.1)	6.8 (2.0,11.5)	4.8 (3.6,6.0)	0.1 (-0.3,0.5)	0.2 (0.03,0.3)	0.01 (-0.3,0.4)	0.2 (0.07,0.4)	0.1 (-0.2,0.5)	0.17 (0.09,0.26)

[a] Adjusted for maternal age, marital status, educational attainment, pre-pregnancy BMI, total gestational weight gain, smoking history, glucose tolerance status, and gestational hypertensive disorders and child gestational age at delivery, race/ethnicity, sex, birth weight for gestational age z-scores, breastfeeding status at 6 months and age at outcome measurement.
[b] Adjusted for maternal age, maternal BMI at 6.5 years, educational attainment, marital status, smoking during pregnancy, gestational age at delivery, child sex, birth weight for gestational age z-scores and age at outcome measurement.

© 2018 Aris IM et al. *JAMA Network Open.*
